# Supplementary material for: Poor sleep quality and its associated factors among pregnant women in Northern Ethiopia, 2020: A cross sectional study
Source: PLoS One. 2021 May 4;16(5):e0250985. doi: 10.1371/journal.pone.0250985 (PMC8096079; doi:10.1371/journal.pone.0250985)
Supplement: S1 Appendix — (DOCX) [file pone.0250985.s001.docx]

**Questionnaire by Original language (Amharic)**

የአማርኛ መጠይቅ

የጥናቱ ተሳታፊ መለያ ቁጥር-------------------------

ክፍል I**.** ማህበራዊና ዲሞግራፊያዊ ሁኔታን የሚመለከቱ ጥያቄዎች

| ተ/ቁ | ጥያቄዎች | ምላሾች |
| --- | --- | --- |
| 101 | ዕድሜ | _______________ዓመት |
| 105 | የትምርት ሁኔታ | 1. ያልተማረች 2. አንደኛ ደረጃ 3. ሁለተኛ ደረጃ 4.  ዲፕሎማና በላይ |
| 106 | የኣሁኑ ወቅት የጋብቻ ሁኔታ | 1. ያላጋባች 2. ያገባች 3. የፈታች 4. ባል የሞተባት |
| 107 | ስራ | 1. የመንግስት ሰራተኛ 2. የግል ሰራተኛ 3. ነጋዴ 4. አርሶ አደር 5. የቤት እመቤት 6. የቀን ሰራተኛ 7.ስራ የሌለው 8. ሌላ ካለ_ |
| 108 | ወርሃዊ ገቢ | ---------------------ብር |
| 109 | በቋሚነት የሚኖሩበት ቦታ | 1. ከተማ 2. ገጠር |

ክፍል II**.** የእርግዝናና የህክምና ታሪክ

| 201 | የእርግዝናዉ እድሜ | **-------------------**ሳምንት |
| --- | --- | --- |
| 202 | የወሊድ ሁኔታ | 1.የመጀመሪያ  2.ከዛ በላይ |
| 203 | ተጨማሪ የደም ግፊት መጨመር አለባቸው? | 1. አዎ 2. የለም |
| 204 | ከእርግዝናው ጋር የተያያ ሌላ ችግር አለ | 1.አወ 2. የለም |
| 205 | BP(convenient arm) SBP-------------mmHg DBP------------mmHg | |
| 207 | የደም ማነስ መከላከያ እንክብል ወስደዋን 1. አዎ 2. የለም | |

ክፍል **III**. የንጥረ ነገር ሁኔታ ጥያቄዎች **(**አልኮሆል መጠጥ፣ ጫት መቃም፣ ሲገራ ማጨስ**)**

| 301 | በሂዎትዎ ቡና ወይም ሻይ ጠጥተው ያውቀሉ? | 0. አዎ 1. አላውቅም |
| --- | --- | --- |
| 302 | ለጥየቄ 301መልሱ አዎ ከሆነ, ባለፈው 30 ቀናት ዉስጥ ጠጥተዋል? | 0. አዎ 1. አላውቅም |
| 303 | ለጥየቄ 302መልሱ አዎ ከሆነ,ምን ያህል ግዜ ይጠቀማሉ | 0.በየቀኑ 2. በየሰምንቱ |
| 304 | በሂዎትዎ ጫት ቅመው ያውቀሉ | 0. አዎ 1. አላውቅም |
| 305 | ለጥየቄ 304 መልሱ አዎ ከሆነ, ለስንት ጊዜ ያህል ቅመወል?(በወር ) | --------------------ወር |
| 306 | ለጥየቄ 304መልሱ አዎ ከሆነ, ባለፉት 30 ቀናት ዉስጥ ቅመወል? | 0. አዎ 1. አላውቅም |
| 307 | ለጥየቄ 306 መልሱ አዎ ከሆነ, ምን ያህል ጊዜ ይቅማሉ? | 0.በየቀኑ 2. በየሰምንቱ |
| 308 | ለጥየቄ 306 መልሱ አዎ ከሆነ, በቀን ምን ያህል መጠን ጫት ይቅማሉ ? | -----------------ግራም(ሁርባ) |
| 309 | በሂዎትዎ አልኮሆል ያለበት መጠጥ ጠጥተው ያውቃሉ? | 0. አዎ 1. አላውቅም |
| 310 | ለጥየቄ 309 መልሱ አዎ ከሆነ, ለስንት ጊዜ አልኮሆል ተጠቅመዋል? | 0 . 6 ወር 1. 1 ዓመት 2. 2ዓመት 3. > 2 ዓመት |
| 311 | ለጥየቄ 309 መልሱ አዎ ከሆነ,ባለፉት 30 ቀናት አልኮሆል ተጠቅመወል? | 0. አዎ 1. አላውቅም |
| 312 | ለጥየቄ 311 መልሱ አዎ ከሆነ, ምን አይነት አልኮሆል ተጠቀሙ?ዘርዝር | ----------፤-----------፤------------ |
| 313 | ለጥየቄ 311 መልሱ አዎ ከሆነ,ምን ያህል አልኮሆል በሳምንት ይጠቀማሉ? | _____ሊ |
| 314 | በሂዎትዎ ሲጋራ አጭሰው ያውቃሉ? | 0. አዎ 1. አላውቅም |
| 315 | ለጥየቄ 314 መልሱ አዎ ከሆነ, ባለፉት 30 ቀናት አጭሰዋል? | 0. አዎ 1. አላውቅም |
| 316 | ለጥየቄ 315 መልሱ አዎ ከሆነ,በቀን ምን ያህል ሲጋራ ያጨሳሉ ?(ቁጥር) | ________ |

ክፍል **III**. የእንቅልፍ ጥራት መለክያ መጠይቅ

መመሪያ 3 ፤ የሚከተሉት ጥያቄዎች ባለፈው ወር ውስጥ የነበርዎት የእንቅልፍ ልማድ ጋር የሚዛመዱ ናቸው፡፡ስለዚ መልስዎ ያለፈው ወር ቀን ና ሌሊት ትክክለኛ የእንቅለፍ ልማድ የሚጠቁሙ ሊሆኑ ይገባል፡፡

1. ያለፈው ወር ውስጥ አብዛኛው ግዜ ወደ መኝታ የምሄዱት ስንት ሰኣት ላይ ነው? ___________

2. ኣልጋ ላይ ከወጡ በኃላ በምን ያህል ደቂቃዎች ውስጥ እንቅልፍዎ ይመጣል? ____________

3. አብዛኛው ግዜ ከእንቅልፍ የሚነቁት ስንት ሰዓት ነው? __________

4. ሀ. በአንድ ለሊት ምን ያህል ሰዓታት ትክክለኛ እንቅለፍ ያገኛሉ? _____________

ለ. በአንድ ለሊት ምን ያህል ሰዓታት ኣልጋ ላይ ይቆያሉ? _______________

| 5 | ያለፈው ወር ውስጥ በሚከተሉት ምክንያት ምን ያህል ግዜ የእንቅልፍ ችግር ኣጋጥሞታ | ያለፈው ወር ወስጥ ኣላጋጠመኝም  (0) | አንዴ በሳምንት (1) | ሁለቴ በ ሳምንተ (2) | ሶስቴ ና ከዛበላይ በ ሳምንት (3) |
| --- | --- | --- | --- | --- | --- |
|  | A. በ 30 ደቂቃ ውስጥ እንቅልፍ ኣይወስደኝም ነበረ |  |  |  |  |
|  | B. ሌሊት ወይም ገና ሳይነጋ ከእንቅለፍ እነቃ ነበረ |  |  |  |  |
|  | C. ሽንት ቤት ለመሄድ እነቃ ነበረ |  |  |  |  |
|  | D. በቀላሉ መተንፈስ ኣልችልም ነበረ |  |  |  |  |
|  | E. ያስለኝ ነበረ/ የማንኮረፋት ችግር ነበረብኝ |  |  |  |  |
|  | F. በከፍተኛ ደረጃ ይበርደኝ ነበረ |  |  |  |  |
|  | g. በከፍተኛ ደረጃ ይሞቀኝ ነበረ |  |  |  |  |
|  | H. መጥፎ ህልም ይታየኝ ነበረ |  |  |  |  |
|  | I. ሕመም ይሰማኝ ነበረ |  |  |  |  |
|  | J. ሌላ ምክንያት ካለ ምን ያህል ግዜ እንቅልፍ ያስቸግርዎት እንደነበረ ያብራሩ |  |  |  |  |

| 6 | ባለፈው ወር ውስጥ ለእንቅልፍ ሊረዳዎት የሚችል መድሃኒት ምን ያህል ገዜ ተጠቅመዋል |  |  |  |  |
| --- | --- | --- | --- | --- | --- |
| 7 | ባለፈው ወር ውስጥ ሲነዱ ፣ ምግብ ሲበሉ ወይም በማህበራዊ እንቅስቃሴ ሲሳተፉ በንቃት እንዳይቆዩ ምን ያህል ገዜ ተቸግረዋል |  |  |  |  |
| 8 | ባለፈው ወር ውስጥ ነገሮች በትጋት ለማከናወን ምን ያህል ያስቸግርዎት ነበረ |  |  |  |  |
|  |  | በጣም ጥሩ  (0) | በኣንፃራዊጥሩ  (1) | በኣንፃራዊ መጥፎ (2) | በጣም መጥፎ (3) |
| 9 | ባጠቃላይ ባለፈው ወር ውስጥ የእርስዎ የእንቅልፍ ጥራት ምን ደረጃ ላይ ነበረ |  |  |  |  |

የይዘት ኣቆጣጠር

| ይዘት | ነጥቦች | ውጤት |
| --- | --- | --- |
| 1 | የ 9 ቁጥር ውጤት | C1____ |
| 2 | የ 2 ቁጥር ውጤት (<15 ደቂቃ (0), 16-30ደቂቃ (1), 31-60 ደቂቃ (2), >60ደቂቃ (3)) + የ5a ቁጥር ውጤት (ድምር ውጤቱ እንደ ሚከተለው ከሆነ 0=0; 1-2=1; 3-4=2; 5-6=3) | C2____ |
| 3 | የ 4ቁጥር ውጤት (>7(0), 6-7 (1), 5-6 (2), <5 (3) | C3____ |
| 4 | (ኣጠቃላይ እንቅልፍ ላይ የቆዩት ሰዓታት) / ((ኣጠቃላይ ኣልጋ ላይ የቆዩት ሰዓታት) x 100 >85%=0, 75%-84%=!, 65%-74%=2, <65%=3 | C4____ |
| 5 | ከ ቁጥር 5b እስከ 5j ድምር ውጤት (0=0; 1-9=1; 10-18=2; 19-27=3) | C5____ |
| 6 | የ 6 ቁጥር ውጤት | C6____ |
| 7 | የ 7 ቁጥር ውጤት + የ 8 ቁጥር ውጤት (0=0; 1-2=1; 3-4=2; 5-6=3) | C7-------- |
