# Supplementary material for: Poor sleep quality and its associated factors among pregnant women in Northern Ethiopia, 2020: A cross sectional study
Source: PLoS One. 2021 May 4;16(5):e0250985. doi: 10.1371/journal.pone.0250985 (PMC8096079; doi:10.1371/journal.pone.0250985)
Supplement: S2 Appendix — (DOCX) [file pone.0250985.s002.docx]

**II –English version questionnaire**

Code number of the study participant ________________

**I. Sociodemographic characteristics of study participants**

| **S/N** | **Variables** | **Answers** |
| --- | --- | --- |
| 101 | Age | ____________ years |
| 102 | Educational status | 1. Illiterate 2. Elementary 3. High school 4. Diploma and above |
| 103 | Marital status | 1. Single 2. married 3.divorced 4.widowed |
| 104 | Occupation | 1. Government employee 2. Private employee  3. Merchant 4. Farmer 5. House wife 6. Daily laborers 7. Without job 8. Others specify------------------ |
| 105 | Monthly Income | _______________________Ethiopian birr |
| 106 | Residence | 1. Urban 2. Rural |

**II. Pregnancy and medical history**

| 201 | Gestational age | | ______ Weeks |
| --- | --- | --- | --- |
| 202 | Parity | | 1. Primigravida  2. Multipara  3. Grand multipara |
| 203 | Presence of co-morbid HTN | | 1. Yes 2. No |
| 204 | Presence of Complication | | 1. Yes ---------------------  2. No |
| 205 | BP(convenient arm) SBP-------------mmHg DBP------------mmHg | | |
| 205 | Iron and foliate supplementation | 1. Yes 2. No | |

**III. Substance use assessment (alcohol intake, khat chewing, and cigarette smoking)**

| S/N | Type of question | Answer |
| --- | --- | --- |
| 301 | Have you ever chewed khat? | 1. Yes 2. No |
| 302 | If yes to Q304, for how many years/months have you chewed khat? | ______months |
| 303 | If yes to Q304, have you chewed khat within the last 30days? | 1. Yes 2. No |
| 304 | If yes to Q306, how often you chew khat? Specify, | 1. Weekly 2. Daily |
| 305 | If yes to Q306, what amount of khat you chew per day? | _______grams |
| 306 | Have you ever drunk alcohol? | 1. Yes 2. No |
| 307 | If yes to Q309, for how long have you been drinking alcohol? | 1. 6 mos. 2. 1 yr.  3. 2 yrs. 4. > 2 yrs |
| 308 | If yes to Q309, have you drink alcohol within the last 30days? | 1. Yes 2. No |
| 309 | If yes to Q311, what type of alcohol do you drink? Specify | ________, _____________, _________________ |
| 310 | If yes to Q311, how much liter of alcohol you drink per week? | ______L |
| 311 | Have you ever smoked cigarette in your life time? | 1. Yes 2. No |
| 312 | If yes to Q314, have you smoked within the last 30days? | 1. Yes 2. No |
| 313 | If yes to Q315, how many cigarettes you smoke daily (in pcs) | ________pcs |
| 315 | If yes to Q314, have you smoked within the last 30days? | 1. Yes 2. No |
| 316 | If yes to Q315, how many cigarettes you smoke daily (in pcs) | ________pcs |

**V. Standardized Sleep quality measurement (PSQI) questions**

**Instructions**: The following questions relate to your usual sleep habits during the past month only. Your answers should indicate the most accurate reply for the majority of days and nights in the past month. Please answer all questions.

During the past month,

1. When have you usually gone to bed? ___________________

2. How long (in minutes) has it taken you to fall asleep each night? _______________

3. When have you usually gotten up in the morning? ___________________

4. A. How many hours of actual sleep do you get at night? (This may be different than the

number of hours you spend in bed) _____________

B. How many hours spend in bed per night? __________

| 5. During the past month, how often have you had trouble Sleeping because you… | Not during the last month(0) | Less than once a week(1) | Once or twice a week (2) | Three or  more  times a week(3) |
| --- | --- | --- | --- | --- |
| a. Cannot get to sleep within 30 minutes |  |  |  |  |
| b. Wake up in the middle of the night or early morning |  |  |  |  |
| c. Have to get up to use the bathroom |  |  |  |  |
| d. Cannot breathe comfortably |  |  |  |  |
| e. Cough or snore loudly |  |  |  |  |
| f. Feel too cold |  |  |  |  |
| g. Feel too hot |  |  |  |  |
| h. Have bad dreams |  |  |  |  |
| i. Have pain |  |  |  |  |
| j. Other reason(s), please describe, including how often you have had trouble sleeping because of this reason(s): |  |  |  |  |
| 6. During the past month, how often have you taken  medicine (prescribed or “over the counter”) to  help you sleep? |  |  |  |  |
| 7. During the past month, how often have you had trouble staying awake while driving, eating meals, or engaging in social activity? |  |  |  |  |
| 8. During the past month, how much of a problem has it been for you to keep up enthusiasm to get things done? |  |  |  |  |
|  | Very  good (0) | Fairly  good(1) | Fairly bad(2) | Very bad(3) |
| 9. During the past month, how would you rate your sleep quality overall? |  |  |  |  |

|  |
| --- |

**Scoring**

Component 1 #9Score......................................................................................C1_______

Component 2 #2 Score (≤15min=0; 16-30 min=1; 31-60 min=2, >60 min=3)

+ #5a Score (if sum is equal 0=0; 1-2=1; 3-4=2; 5-6=3).........C2_______

Component 3 #4 Score (>7=0; 6-7=1; 5-6=2; <5=3).......................................C3_______

Component 4 (total # of hours asleep)/(total # of hours in bed) x 100>85%=0,

75%-84%=1, 65%-74%=2, <65%=3......................................C4_______

Component 5 Sum of Scores #5b to #5j (0=0; 1-9=1; 10-18=2; 19-27=3)...C5_______

Component 6 #6 Score...................................................................................C6_______

Component7 #7Score+#8 Score (0=0; 1-2=1; 3-4=2; 5-6=3)......................C7_______

Add the seven component scores together ________ Global PSQI Score ________
