# Supplementary material for: Poor sleep quality and its associated factors among pregnant women in Northern Ethiopia, 2020: A cross sectional study
Source: PLoS One. 2021 May 4;16(5):e0250985. doi: 10.1371/journal.pone.0250985 (PMC8096079; doi:10.1371/journal.pone.0250985)
Supplement: Attachment — Submitted filename: PONE-D-20-34586_to be reviewed.pdf [file pone.0250985.s004.pdf]

PLOS ONE

Poor sleep quality and its associated factors among pregnant women in Northern Ethiopia, 2020.

--Manuscript Draft--

Manuscript Number:	PONE-D-20-34586
Article Type:	Research Article
Full Title:	Poor sleep quality and its associated factors among pregnant women in Northern Ethiopia, 2020.
Short Title:	Poor sleep quality among pregnant women
Corresponding Author:	Tadeg Jemere, MSc<br>Debre Tabor University<br>Debre Tabor, Amhara ETHIOPIA
Keywords:	Sleep quality, Pregnant mothers, Wadila primary hospital, Ethiopia
Abstract:	Abstract<br><br>Background- Sleep is a physiologic necessity for mammals, as prolonged complete sleep deprivation results in death. A change in sleep quality and quantity is the most common phenomena during pregnancy due to mechanical and hormonal factors. However, there is a scarcity of data about poor sleep quality and its associated factors among pregnant mothers in Ethiopia. Therefore, this study aims to determine the prevalence of poor sleep quality and its associated factors among pregnant mothers at Wadila primary hospital, Ethiopia.<br>Methods: Institution based cross-sectional study design was employed on 411 pregnant mothers. Data were collected using a pre-tested interviewer administered questionnaire. SPSS Version 23 for Windows software was used for summarization and analyses of data. Bivariate analysis was conducted to detect the association between dependent and independent variables, and candidate variables (p < 0.25) for multivariate logistic regression were selected. Statistical significance was set at p-value <0.05.<br>Results: A total of 411 participants were included in the study making a response rate of 97.4 %. Overall, 68.4 % of participants found to have poor sleep quality (PSQI>5). Age of the mother [age 20-30 years; AOR= 4.3 CI (1.8, 9.9), p= 0.001, and age >30 years; AOR= 4.7 CI (1.6, 13.9) p= 0.005], gestational age [first trimester, AOR= 2.46, CI (1.2, 4.9), p= 0.01 and second trimester, AOR= 7.5, CI (3.2, 17.8), p= 0.000] and parity [multiparous women; AOR= 2.1(1.24, 3.6) p=0.006] were predictor variables for poor sleep quality among pregnant mothers.<br>Conclusion: More than two-thirds of pregnant mothers had poor sleep quality. Poor sleep quality may bring about the mother's mental impairment, and the offspring are predisposed to various anxiety disorders and learning disabilities. Therefore, pregnant mothers need to have health education about risk factors of poor sleep quality and sleep hygiene practice.
Order of Authors:	Tadeg Jemere, MSc
	Berhanu Geatahun
	Fitalew Tadele
	Belayneh Kefale
	Gashaw Walle
Additional Information:	
Question	Response
**Financial Disclosure**<br><br>Enter a financial disclosure statement that describes the sources of funding for the	No specific funding for this research

Powered by Editorial Manager® and ProduXion Manager® from Aries Systems Corporation

work included in this submission. Review the submission guidelines for detailed requirements. View published research articles from *PLOS ONE* for specific examples.<br><br>This statement is required for submission and **will appear in the published article** if the submission is accepted. Please make sure it is accurate.<br><br>**Unfunded studies**<br>Enter: *The author(s) received no specific funding for this work.*<br><br>**Funded studies**<br>Enter a statement with the following details:<br>• Initials of the authors who received each award<br>• Grant numbers awarded to each author<br>• The full name of each funder<br>• URL of each funder website<br>• Did the sponsors or funders play any role in the study design, data collection and analysis, decision to publish, or preparation of the manuscript?<br>• **NO** - Include this sentence at the end of your statement: *The funders had no role in study design, data collection and analysis, decision to publish, or preparation of the manuscript.*<br>• **YES** - Specify the role(s) played.<br><br>* typeset	
**Competing Interests**<br><br>Use the instructions below to enter a competing interest statement for this submission. On behalf of all authors, disclose any competing interests that could be perceived to bias this work—acknowledging all financial support and any other relevant financial or non-financial competing interests.<br><br>This statement **will appear in the published article** if the submission is accepted. Please make sure it is accurate. View published research articles from *PLOS ONE* for specific examples.	There is no competing interests

Powered by Editorial Manager® and ProduXion Manager® from Aries Systems Corporation

**NO authors have competing interests**<br><br>Enter: *The authors have declared that no competing interests exist.*<br><br>**Authors with competing interests**<br><br>Enter competing interest details beginning with this statement:<br><br>*I have read the journal's policy and the authors of this manuscript have the following competing interests: [insert competing interests here]*<br><br>* typeset	
**Ethics Statement**<br><br>Enter an ethics statement for this submission. This statement is required if the study involved:<br><br>• Human participants<br>• Human specimens or tissue<br>• Vertebrate animals or cephalopods<br>• Vertebrate embryos or tissues<br>• Field research<br><br>Write "N/A" if the submission does not require an ethics statement.<br><br>General guidance is provided below. Consult the submission guidelines for detailed instructions. **Make sure that all information entered here is included in the Methods section of the manuscript.**	Ethical approval was obtained from ethical review Committee of Debre Tabor University with ethical approval number of DTU/4/258/2020.

Powered by Editorial Manager® and ProduXion Manager® from Aries Systems Corporation

**Format for specific study types**<br><br>**Human Subject Research (involving human participants and/or tissue)**<br>• Give the name of the institutional review board or ethics committee that approved the study<br>• Include the approval number and/or a statement indicating approval of this research<br>• Indicate the form of consent obtained (written/oral) or the reason that consent was not obtained (e.g. the data were analyzed anonymously)<br><br>**Animal Research (involving vertebrate animals, embryos or tissues)**<br>• Provide the name of the Institutional Animal Care and Use Committee (IACUC) or other relevant ethics board that reviewed the study protocol, and indicate whether they approved this research or granted a formal waiver of ethical approval<br>• Include an approval number if one was obtained<br>• If the study involved *non-human primates*, add *additional details* about animal welfare and steps taken to ameliorate suffering<br>• If anesthesia, euthanasia, or any kind of animal sacrifice is part of the study, include briefly which substances and/or methods were applied<br><br>**Field Research**<br><br>Include the following details if this study involves the collection of plant, animal, or other materials from a natural setting:<br>• Field permit number<br>• Name of the institution or relevant body that granted permission	
**Data Availability**<br><br>Authors are required to make all data underlying the findings described fully available, without restriction, and from the time of publication. PLOS allows rare exceptions to address legal and ethical concerns. See the PLOS Data Policy and FAQ for detailed information.	Yes - all data are fully available without restriction

A Data Availability Statement describing where the data can be found is required at submission. Your answers to this question constitute the Data Availability Statement and **will be published in the article**, if accepted.<br><br>**Important:** Stating 'data available on request from the author' is not sufficient. If your data are only available upon request, select 'No' for the first question and explain your exceptional situation in the text box.<br><br>Do the authors confirm that all data underlying the findings described in their manuscript are fully available without restriction?	
**Describe where the data may be found in full sentences. If you are copying our sample text, replace any instances of XXX with the appropriate details.**<br><br>• If the data are **held or will be held in a public repository**, include URLs, accession numbers or DOIs. If this information will only be available after acceptance, indicate this by ticking the box below. For example: *All XXX files are available from the XXX database (accession number(s) XXX, XXX.).*<br>• If the data are all contained **within the manuscript and/or Supporting Information files**, enter the following: *All relevant data are within the manuscript and its Supporting Information files.*<br>• If neither of these applies but you are able to provide **details of access elsewhere**, with or without limitations, please do so. For example:<br><br>*Data cannot be shared publicly because of [XXX]. Data are available from the XXX Institutional Data Access / Ethics Committee (contact via XXX) for researchers who meet the criteria for access to confidential data.*<br><br>*The data underlying the results presented in the study are available from (include the name of the third party*	data are all contained within the manuscript

and contact information or URL).<br>• This text is appropriate if the data are owned by a third party and authors do not have permission to share the data.<br><br>* typeset	
Additional data availability information:	

Powered by Editorial Manager® and ProduXion Manager® from Aries Systems Corporation

Manuscript Click here to access/download;Manuscript;Sleep manuscript.pdf

Poor sleep quality and its associated factors among pregnant women in Northern Ethiopia, 2020.

Tadeg Jemere[1*], Berhanu Getahun[2], Fitalew Tadele[1], Belayneh Kefale[3] and Gashaw Walle[1]

1Department of Biomedical Sciences, College of Health Sciences, Debre Tabor University, Debre Tabor, Amhara, Ethiopia

2Integrated emergency surgery and gyn/obs professional specialist, Amdework primary hospital, Amdework, Wollo, Ethiopia

3Clinical Pharmacy Unit and Research team, Department of Pharmacy, College of Health Sciences, Debre Tabor University, Debre Tabor, Amhara, Ethiopia

*Corresponding Author: Tadeg Jemere

Medical Physiology Unit, Department of Biomedical Sciences, College of Health Sciences, Debre Tabor University, Debre Tabor, Amhara, Ethiopia

Email address: - tadegamare12@gmail.com

Abstract

Background- Sleep is a physiologic necessity for mammals, as prolonged complete sleep deprivation results in death. A change in sleep quality and quantity is the most common phenomena during pregnancy due to mechanical and hormonal factors. However, there is a scarcity of data about poor sleep quality and its associated factors among pregnant mothers in Ethiopia. Therefore, this study aims to determine the prevalence of poor sleep quality and its associated factors among pregnant mothers at Wadila primary hospital, Ethiopia.

Methods: Institution based cross-sectional study design was employed on 411 pregnant mothers. Data were collected using a pre-tested interviewer administered questionnaire. SPSS Version 23 for Windows software was used for summarization and analyses of data. Bivariate analysis was conducted to detect the association between dependent and independent variables, and candidate variables ($p < 0.25$) for multivariate logistic regression were selected. Statistical significance was set at p-value <0.05.

Results: A total of 411 participants were included in the study making a response rate of 97.4 %. Overall, 68.4 % of participants found to have poor sleep quality (PSQI>5). Age of the mother [age 20-30 years; AOR= 4.3 CI (1.8, 9.9), *p*= 0.001, and age >30 years; AOR= 4.7 CI (1.6, 13.9) *p*= 0.005], gestational age [first trimester, AOR= 2.46, CI (1.2, 4.9), p= 0.01 and second trimester, AOR= 7.5, CI (3.2, 17.8), p= 0.000] and parity [multiparous women; AOR= 2.1(1.24, 3.6) p=0.006] were predictor variables for poor sleep quality among pregnant mothers.

Conclusion: More than two-thirds of pregnant mothers had poor sleep quality. Poor sleep quality may bring about the mother's mental impairment, and the offspring are predisposed to various anxiety disorders and learning disabilities. Therefore, pregnant mothers need to have health education about risk factors of poor sleep quality and sleep hygiene practice.

Key words: sleep quality, pregnant mothers, Wadila primary hospital, Ethiopia

Introduction

Sleep is a physiologic necessity for mammals, as prolonged complete sleep deprivation results in death (1). The prevalence of sleep disturbances and deprivation has been increasing dramatically over the past decade (2). A change in sleep quality and quantity is a common phenomenon during pregnancy (1). The total sleep time varies through pregnancy, with an increased total sleep time in the 1st trimester of pregnancy, normalized sleep time in the 2nd trimester and decreased sleep time in the 3rd trimester. More frequent or prolonged awakenings are observed with increasing gestational age. Sleep disorders such as subjective snoring, sleep disordered breathing and restless legs syndrome are also known to increase in prevalence during pregnancy (3,4).

Sleep can be altered during pregnancy due to mechanical and hormonal factors. This may be due to physical and mental changes that the body undergoes during pregnancy. Problems such as leg cramps, urinary incontinence, shortness of breath, heart burn, and intense backaches are among the mechanical factors. Some women may also have difficulty in finding the right position to sleep because of the growing uterus, a very active baby, and worries about the baby and themselves (5,6).

There is also a marked increase in the level of estrogen and progesterone during pregnancy. Increasing evidence suggests that, these hormones control reproductive functions and influence a diverse range of physiological and psychological processes, including sleep, mood, sensory, and cognitive functions (7). The level of estrogen is elevated more than 100 times and progesterone up to 200 times in late pregnancy compared to pre-pregnancy level. Estrogen causes edema of the upper airway mucosa, leading to increased upper airway resistance and difficulty in breathing (4). Oxytocin, the hormone responsible for uterine contractions, is known to peak at night, possibly causing sleep fragmentation in late pregnancy (8).

Prevalence rates for altered sleep quality during pregnancy range from 40% to 96%, depending on the population studied and the time of assessment being higher during the third trimester of pregnancy (9–13). According to the result of different studies, increased age, advanced

education, employment status, monthly income, gestational age, parity, alcohol consumption and smoking are found to be factors affecting the sleep quality of pregnant women (3,5,14,15).

Poor sleep quality may bring about mental impairment of the mother and fetal neural networks are highly vulnerable to maternal sleep loss. The offspring are predisposed to various anxiety disorders and learning disabilities (10). Though the prevalence and factors affecting poor sleep quality among pregnant mothers have been investigated in developed world, this is not well understood in developing countries like Ethiopia. Hence, this study aims to determine the prevalence of poor sleep quality and its associated factors among pregnant mothers.

Materials and Methods

Study design, area and period

Institution based cross-sectional study design was employed at Wadila primary hospital, Kone town. Kone town is located in Amhara regional state, North Wollo zone, 623 kilometers north of Addis Ababa, Ethiopia. Wadila primary hospital is one of the youngest hospitals in Ethiopia and providing service for approximately 200,000 people in the catchment area, including antenatal care (ANC) service for pregnant mothers. The data were collected from May 15 to August 15, 2020.

Source and study population

All pregnant mothers who came to the hospital for ANC follow up were source populations and all pregnant mothers who came to the hospital for ANC follow up and present at the time data collection were study populations.

Eligibility criteria

All pregnant mothers who came to the hospital for ANC follow up and present at the time of data collection were included. Whereas, pregnant mothers who had established psychiatric disorder or use any kind of psychotropic medication, chronic illnesses like asthma, heart failure, and cancer, and who work in night shifts in the last month were excluded.

Sample size determination and sampling technique

Sample size determination

The sample size was determined using the statistical formula for single population proportion by considering the proportion of poor sleep quality as 50% (since no study is conducted in Ethiopia), with a 95% confidence interval, 5% margin of error, and with the assumption of 10% non-response rate. Hence, the final sample size was 422. Study participants were selected using systematic random sampling technique.

Data collection procedure

Data were collected using a pre-tested interviewer administered questionnaire which contains four parts. These includes: sociodemographic and substance use-related variables, medical record reviews and pregnancy-related variables, a validated standardized test for sleep quality, the Pittsburgh Sleep Quality Index (PSQI) (16), and blood pressure (BP) measurement.

Blood pressure was measured using an automated digital BP monitor complete with adult cuff and participants in sitting position with the arm at the heart level and after 5 minutes rest.

The PSQI is a 19-item self-report measure designed to measure sleep quality over the past month. The PSQI has seven subscale scores that measure subjective quality, latency, duration, habitual sleep efficiency, disturbance, medication use, and daytime dysfunction as they relate to sleep. These subscales are added to determine a global sleep quality score (GSQ). The GSQ score ranges from 0 to 21, in which higher scores (PSQI >5) indicate poor sleep quality (16).

Data were collected by two BSc midwives who had previous data collection experience, supervised by the principal investigator (PI). Training was given to data collectors by the PI before data collection starts. A week before the actual time of data collection, the questionnaire was tested on 5% of the participants having similar sociocultural characteristics with the study participants at Kone health center.

Operational Definitions

Good sleep quality is a state of having PSQI score ≤5 and individuals who had PSQI score >5 were said to have poor sleep quality (16). Pregnant mothers who use any of the substances such as alcohol, khat, and cigarette smoking at least once in their life time were classified as ever user. Participants who consumed psycho-stimulant substances such as alcohol, khat, and cigarette smoking at least once within the last 30 days were classified as current user.

Data Analysis Procedures

The collected data were checked for completeness and coded then entered into Epi-data version 3.1. SPSS Version 23 for Windows software was used for summarization and analyses of data. Frequency, mean and proportions were used for the descriptive analysis of data. Bivariate analysis was conducted to detect the association between dependent and independent variables, and candidate variables ($p < 0.25$) for multivariate logistic regression were selected. We used $p < 0.25$ as a cutoff point to select candidate variables of the final model so as to improve the chances of retaining meaningful confounders. Odds ratio and its 95% confidence interval were estimated for potential predictors of poor sleep quality, which were included in the final model. Statistical significance was set at p-value <0.05.

Data Quality Management

To assure data quality, pre-tested interviewer administered questionnaire was used to collect data. A 2 day training was given for data collectors regarding the purpose of the study and measurement techniques. The questionnaire was translated to Amharic language and then retranslated back to English to maintain its consistency. To minimize bias, respondents were assured to keep their response confidential.

Results

Sociodemographic characteristics

A total of 411 participants were included in the study making a response rate of 97.4 %. The majorities (71.3 %) were in the age range of 20-30 years with the mean age of 25.7 ± 5.5. Around 30 % of participants did not attend formal education and 28.2 % attend elementary education. Majorities (94.9%) were married and the lowest percentages were widowed (1.7 %). More than half (52.8%) were housewives and 19 % of them were government employees. From all participants, 52.1 % were rural residents and the average monthly income of the participants was 1775.7 ± 1610 (Table 1).

Pregnancy related characteristics

More than half (54.3 %) of the participants were in the second trimester of pregnancy and the lowest percentages (12.2 %) were in the first trimester of pregnancy. On the other hand, 65.5 % of participants were multiparous and the rest 34.5% were nulliparous. From all participants, only 4.1 % had history of still birth and 7.5 % had history of abortion. The majority (76.9 %) of

participants received iron and folic acid supplementation. Regarding their blood pressure, 6.1 % of participants had increased blood pressure at the time of data collection.

Table 1: Sociodemographic characteristics of study participants at Wadila primary hospital, Wadila, Northern Ethiopia, 2020.

Study groups n=198			
Variables	Category	Number of participants	Frequency %
Age	<20	52	12.7
	20-30	293	71.3
	>30	66	16
Educational status	No formal education	123	29.9
	Primary	116	28.2
	Secondary	91	22.1
	≥Diploma	81	19.8
Marital status	Single	14	3.4
	Married	390	94.9
	Divorced	0	-
	Widowed	7	1.7
Occupation	Employed	89	21.7
	Merchant	42	10.2
	Farmer	45	10.9
	House wife	205	50.4
	Others*	28	6.8
Residence	Urban	197	47.9
	Rural	214	52.1
Monthly income (ETB)	<1000	154	37.5
	1000-4000	234	56.9
	>4000	23	5.6

Substance use profile of study subjects

As per the current study result, all participants (100 %) did not chew khat and smoke cigarette even at least once in their life time. Regarding alcohol consumption, (37.2%) of participants found to have history of alcohol consumption and 17 % of them currently practiced drinking alcohol.

Prevalence of Sleep Quality

The overall prevalence of poor sleep quality was 68.4 %, at 95% CI (63.7, 72.7) with a mean GSQ score of 6.17 (SD $\pm$ 2.7). The average bed time of the participants was at 9:10 pm, whereas the average wake up time was 6:45 am. The mean total time to fall asleep was 42 minutes (SD +

27 minutes) and the actual sleep time ranges from 2 up to 10 hours with a mean of 6.7 hours (SD +1.6) as reported on the PSQI.

The seven components of sleep quality were assessed using the PSQI and accordingly only one (0.2%) participant rate her subjective sleep quality very bad and 87.1 % of pregnant mothers had ≤7 hours of sleep per night. On the other hand, no participant reports the use of sleep medication during the last month (a month before data collection). Moreover, the habitual sleep efficiency was ≥85% in 19.2% and <65% in 17.5% of the study participants (Table 2).

Table 2: Sleep quality and its component scores among study participants at Wadila primary hospital, Wadila, Northern Ethiopia, 2020.

Study participants (n=411)			
Variables	Values	Number of participants	Frequency (%)
Sleep duration	>7	123	29.9
	6-7	187	45.5
	5-6	54	13.2
	<5	74	11.4
Sleep latency	Never (0)	53	12.9
	<1times a week (1)	121	29.4
	1-2 times a week (2)	168	40.9
	≥3 times a week (3)	69	16.8
Sleep efficiency	≥85%	79	19.2
	75-84%	157	38.2
	65-74%	103	25.1
	<65%	72	17.5
Day time dysfunction	Never (0)	278	67.6
	<1times a week (1)	125	30.4
	1-2 times a week (2)	7	1.7
	≥3 times a week (3)	1	0.2
Sleep disturbance	Never (0)	17	4.1
	<1times a week (1)	376	91.5
	1-2 times a week (2)	18	4.4
	≥3 times a week (3)	0	0
Use of sleep medication	Never (0)	411	100
	<1times a week (1)	0	0
	1-2 times a week (2)	0	0
	≥3 times a week (3)	0	0
Subjective sleep quality	Very good (0)	122	29.7
	Fairly good (1)	261	63.5
	Fairly bad (2)	27	6.6
	Very bad (3)	1	0.2

Factors independently associated with poor sleep quality

From the total variables included in the backward logistic regression model, three variables were found to be statistically significant (*p*<0.05) with 36.7% fitness of model summary (Hosmer and Lemeshow test). Accordingly, age [age 20-30 years; AOR= 4.3 CI (1.8, 9.9), *p*= 0.001, and age >30 years; AOR= 4.7 CI (1.6, 13.9) *p*= 0.005], gestational age [first trimester, AOR= 2.46, CI (1.2, 4.9), p= 0.01 and second trimester, AOR= 7.5, CI (3.2, 17.8), p= 0.000] and parity [multiparous women; AOR= 2.1(1.24, 3.6) p=0.006] were identified to have statistically significant association with poor sleep quality (Table 3).

Table 3: Bivariable and multivariable logistic regression model of factors independently associated with poor sleep quality among pregnant mothers at Wadila primary hospital, Wadila, Northern Ethiopia, 2020, (N=411).

	Poor sleep quality		OR (95% CI)			
Variables	Yes N (%)	No N (%)	COR (95% CI)	*p*-value	AOR (95% CI)	*p*-value
Age <20	22(42.3	30(57.7)	1		1	
20-29	204(69.6)	89(30.4)	3.13(1.7, 5.7)	**0.00**	4.3(1.8, 9.9)	**0.001***
≥30	55(83.3)	11(16.7)	6.8(2.9, 15.9)	**0.00**	4.7(1.6, 13.9)	**0.005***
Monthly income						
<1000	97(63)	47(37)	1.3(0.539,3.178)	0.552	1.3(0.5, 3.6)	0.642
1000-4000	171(73.1)	63(26.9)	2.1(0.872, 5.001)	**0.099**	2.4(1.3, 6.2)	0.066
>4000	13(56.5)	10(43.5)	1		1	
Gestational age						
First trimester	25(50)	25(50)	1		1	
Second trimester	146(65.5)	77(34.5)	1.9(1.021, 3.522)	**0.043**	2.46(1.2, 4.9)	**0.010***
Third trimester	110(79.7)	28(20.3)	3.9(1.966, 7.851)	**0.00**	7.5(3.2, 17.8)	**0.000***
Parity						
Nulliparous	76(53.5)	66(46.5)	1		1	
Multiparous	205(76.2)	64(23.8)	2.8(1.8, 4.289))	**0.000**	2.1(1.24, 3.6)	**0.006***

* Statistically significant at *p*-value <0.05; COR: Crude Odds Ratio; AOR: Adjusted Odds Ratio

Discussion

Sleep is a physiological need for all human kind. Pregnant women, in particular, need adequate sleep to develop their fetuses as well as save energy required for delivery (9). Pregnancy is a process that creates significant anatomical, physiological, and biochemical changes in a woman's life (5). These changes affect the physical and emotional behaviours of women and may lead to sleep disturbances. Poor sleep quality can result in pre-term birth, gestational hypertension and cesarean section deliveries (3,17).

The results of this study indicate that women experience significant sleep problems during pregnancy. One of the most common problems experienced by pregnant women was frequent night wakings (92.5 %). From the total participants 68.6 % of women experienced difficulty falling asleep within 30 minutes and waking to use toilet. More than a quarter (27.7 %) of the women also found to have subjective snoring problem during pregnancy. These findings were supported by other studies conducted in Vietnam (18), China (17) and India (19).

On the other hand, 29.7 % of pregnant mothers rate their sleep quality very good, 87.1 % of study participants had ≤7 hours of sleep per night and no participant reports the use of sleep medication during the last month. More ever, the habitual sleep efficiency was ≥85% in 19.2% and <65% in 17.5% of the study participants. This finding is also similar to other studies conducted in Denmark (20), Iran (21), and Vietnam (18). Mechanical factors like frequent urination, very active baby, heart burn and hormonal factors like, increased level of estrogen and oxytocin might be responsible for increased sleep disturbance during pregnancy (4–6,8).

According to this study, the overall prevalence of poor sleep quality was 68.4%. This is in line with the studies conducted in China (64%) (22) and Malaysia (69.4%) (13). However, our finding is lower than the findings of another similar study in USA (76%) (23), Iran (77%) (9), Indonesia (78.9%) (24), Turkey (86%) (14), and Korea (96.2%) (11). The results of this study is higher than studies conducted in Brazil, 56.3% (25), Vietnam, 41.2% (18) and Pakistan, 53.3% (26). The possible reason for this discrepancy may be the difference in PSQI cut of point, sample size, study setting and socioeconomic status of the populations.

Maternal age was identified as a predictor of poor sleep quality in our study, suggesting that sleep quality of pregnant mothers declines as their age increases. This was in agreement with the results of studies conducted in China (17) and Turkey (14). As a potential high-risk factor, women with advanced age were more likely affected by physiological and psychological factors, thus leading to the deterioration of sleep quality (17).

A significant association between gestational age and poor sleep quality was identified among pregnant mothers in this study, showing that general sleep quality declines as pregnancy proceeds. When compared with the first trimester of pregnancy, significant deteriorations in

sleep quality were observed in the second and third trimester of pregnancy. This is similar with the findings of studies conducted in Turkey (5), Pakistan (26) and China (17). This might be due to the increment of hormonal changes, maternal stress and fetal movement as gestational age increases.

Another variable that predicts the occurrence of poor sleep quality among pregnant women in this study was parity. In the present study, multiparous women were 2.1 times more likely to have poor sleep quality than nulliparous women. This is in agreement with the result of the studies conducted in Vietnam (18) and USA (27). The possible justification could be due to the fact that multiparous women are likely to have a young child in the home and their total sleep duration and sleep efficiency are likely to be affected by childcare demands and children's sleep schedules, including nighttime wakings (27).

Limitation of the study

Due to the cross-sectional nature of the study, a causal relation cannot be established and sample size was limited. Despite these limitations, our study clearly showed the magnitude of poor sleep quality and its predictors in our country, which is not well investigated so far.

Conclusions

Sleep disorder is a common problem for pregnant women in Ethiopia, with a prevalence of 68.4%. Furthermore, increased age, gestational age and multiparty are found to be determinants of poor sleep quality in pregnant women. Therefore, we recommend that pregnant mothers need to have health education about risk factors of poor sleep quality and sleep hygiene practice.

Ethics approval and consent to participate

Ethical approval was obtained from ethical review Committee of Debre Tabor University with ethical approval number of DTU/4/258/2020. Written informed consent was taken from each study participants. Confidentiality of information was kept properly.

Consent for publication

Not applicable

Availability of data and material

The authors confirm that all data underlying the findings are fully available without restriction. All relevant data are within the manuscript.

Competing interests

The authors declared that they have no competing interests in this work.

Funding

There is no specific funding for this study.

Author's Contributions

TJ & BG: designed the study, collected data, analyzed it, interpret the result and prepare the manuscript for publication. FT: Conceived the study, supervise the data collection, reviewed the proposal and draft of the manuscript. BK: Participated in designing the study, supervision of the research project and review the draft of the manuscript. GW: Participated in designing the study, analyzed the result and prepared the manuscript for publication. All authors read and approve the manuscript.

Acknowledgments

We would like to express our appreciation to Debre Tabor University for its support to do this research project. We would like to thank our study participants and data collectors for their willingness and cooperation.

References

1. Silvestri R, Aricò I. Sleep disorders in pregnancy. Sleep Sci. 2019;12(3):232–9.
2. Vgontzas AN, Kales A. SLEEP AND ITS DISORDERS. SLEEP Disord. 1999;50(1):387–400.
3. Reichner CA. Insomnia and sleep deficiency in pregnancy. Obstet Med. 2015;8(4):168–71.
4. Sharma S and Franco R. Sleep and Its Disorders in Pregnancy. Wis Med J. 2004;103(5):48–52.
5. Sut HK, Asci O, Topac N. Sleep Quality and Health-Related Quality. J Perinat Neonat Nurs. 2016;34(4):302–9.
6. Abd A, Ayoub EG, Farahat H. The Relationship between Sleep Disturbance in Late Pregnancy and Labor Outcomes. OSR-JNHS. 2016;5(6):174–80.
7. Deurveilher S, Rusak B, Semba K. Female Reproductive Hormones Alter Sleep Architecture in Ovariectomized Rats. LEEP. 2011;34(4):519–30.
8. Oyiengo D, Louis M, Hott B, Bourjeily G. Sleep disorders in Pregnancy. Clin Chest Med

[Internet]. 2014; Available from: http://dx.doi.org/10.1016/j.ccm.2014.06.012

9. Ahmadi Z, Bakouei F, Bakhtiari A. Maternal sleep quality in late pregnancy: The association between preterm birth and sleep quality. Casp J Reprod Med. 2019;5(1):17–22.

10. Facco FL, Kramer J. Sleep Disturbances in Pregnancy. Obstet Gynecol. 2010;115(1):13–5.

11. Ko H, Shin J, Kim MY, Kim YH, Lee J, Kil KC, et al. Sleep disturbances in Korean pregnant and postpartum women. J Psychosom Obstet Gynecol. 2012;33(2):85–90.

12. In SEJ, In P, Mansouri A, Bahrami-vazir E, Mohammad-alizadeh-charandabi S. The Relationship Between Leg Muscle Cramps and Sleep Quality in Pregnant Women Visited the Health Centers in Tabriz The Relationship Between Leg Muscle Cramps and Sleep Quality in Pregnant Women Visited the Health Centers in Tabriz. Shiraz E-Med J. 2019;(June):1–7.

13. Chuin A, Teong A, Diong AX, Omar SZ, Tan PC. The Impact of Self-Reported Sleep on Caesarean Delivery in Women Undergoing Induction of Labour : A Prospective Study. Sci Rep [Internet]. 2017;(September):1–8. Available from: http://dx.doi.org/10.1038/s41598-017-12410-7

14. INihal TASKIRAN. Pregnancy and sleep quality. Turk Soc Obs Gynecol. 2011;8(3):181–7.

15. Lee C. Factors Associated With Sleep Quality in Pregnant Women. Nurs Res. 2011;60(6):405–12.

16. Buysse, D.J., Reynolds III, C.F., Monk, T.H., Berman, S.R., & Kupfer DJ. The Pittsburgh Sleep Quality Index: A new instrument for psychiatric practice and research. J Psychiatr Res. 1989;28(2):193–213.

17. Yang Y, Mao J, Ye Z, Li J, Zhao H, Liu Y. Determinants of sleep quality among pregnant women in China : A cross-sectional survey. Nurs Palliat Care. 2017;2(3):1–5.

18. Thi N, Huong T, Thi N, Thuy H, Thi L, Yen H. Quality of Sleep among Pregnant Women. Int J Clin Med. 2019;10:16–25.

19. Venugopal L, Rajendran P, Parghavi V. A study on assessment of sleep quality in south Indian pregnant women. Int J Res Med Sci. 2018;6(10):3197–201.

20. Ertmann RK, Nicolaisdottir DR, Kragstrup J, Siersma V, Lutterodt MC. Sleep complaints

in early pregnancy . A cross-sectional study among women attending prenatal care in general practice. BMC Pregnancy Childbirth. 2020;6:1–9.

21. Rezaei E, Sc M, Moghadam ZB, Ph D, Saraylu K, Sc M. Quality of Life in Pregnant Women with Sleep Disorder. J Fam Reprod Heal. 2013;7(2):87–93.
22. Xie XH, Wang Y, Huang XX SH. Investigation of the status of sleep quality in hospitalized preterm labour women. J Nurs Admin. 2012;12:397–405.
23. Cheng P, Swanson LM, Palagini L, Bazan LF, Roth T, Drake CL. Insomnia , Short Sleep , And Snoring In Mid-To-Late Pregnancy : Disparities Related To Poverty , Race , And Obesity. Nat Sci Sleep. 2019;11:301–15.
24. Astuti Y. The Factors Influencing Sleep Quality of Pregnant Women in Yogyakarta , Indonesia. Adv Heal Sci Res. 2019;15:203–6.
25. Patrícia V, Sousa S De, Ribeiro SO, Monique C, Aquino R De. Quality of sleep in pregnant woman with low back pain. Fisioter Mov. 2015;28(2):319–26.
26. Ahmed N, Gynaec B, Ahmed N, Khan A, Waseem A, Khan T, et al. Prevalence of Sleep Disturbances During Pregnancy – A Pilot Study. BAOJ Gynaec. 2019;2(3):1–8.
27. Christian LM, Carroll JE, Porter K, Hall MH. Sleep quality across pregnancy and postpartum : effects of parity and race. Sleep Heal J Natl Sleep Found [Internet]. 2019;https://do:1–8. Available from: https://doi.org/10.1016/j.sleh.2019.03.005
