# Supplementary material for: Poor sleep quality and its associated factors among pregnant women in Northern Ethiopia, 2020: A cross sectional study
Source: PLoS One. 2021 May 4;16(5):e0250985. doi: 10.1371/journal.pone.0250985 (PMC8096079; doi:10.1371/journal.pone.0250985)
Supplement: Attachment — Submitted filename: reveiew report for plos one manuscript.docx [file pone.0250985.s003.docx]

| Title | **LECTURER** |
| --- | --- |
| Name | **AUMA ANNA GRACE** |
| Email | **annakado4@gmail.com** |
| Affiliation | **LIRA UNIVERSITY** |
| Country | **UGANDA** |
| Specialization | **MATERNAL, NEONATAL, CHILD AND ADOLESCENT HEALTH (MIDWIFERY)** |

**REVIEW REPORT**

**Reviewer’s information**

**Manuscript Information**

| Manuscript Number | PONE-D-20-34586 |
| --- | --- |
| Manuscript Title | Poor sleep quality and its associated factors among pregnant women in Northern, Ethiopia, 2020 |
|  |  |
|  |  |

**Evaluation Report Per Manuscript Sections**

| General Comment | It is a new addition to the body of knowledge; it is not so much studied and there is a lot of gray area to be explored.  However, the title could include the study design too. | **Page number** |
| --- | --- | --- |
| Abstract | **In your conclusion, you indicated that** the offspring are predisposed to various anxiety disorders and learning disabilities as a result of poor sleep quality, is this from the study result? If so, please indicate how you measured it  Wadila primary hospital is a study site and is not necessary in the list of the key words. | **On page 02** |
| Introduction | Introduction is well stated, although will need to be aligned from global perspective, regional to the nation and the local back ground of the subject.  Consider including relevant controversies around this field  Check if the aim of the study was achieved in this particular paper and include in the background statement. | **On pages 3 and 4** |
| Methodology | **Study method is well aligned, sample size adequate**  **Data collection procedure adequately explained**  **However,**  **Indicate how a systematic random sampling got you to 422 samples**    **Clearly state the statistical significance of the variables on the methodology** | **On pages, 4 and 5** |
| Results | **Results have been presented logically and chronologically,**  **Yu could make the table invisible then** | **On pages 6, 7, and 8** |
| Discussion | **The discussion is well brought out; however, it does don urge out what other studies have found, what it means to this study and why the similarities or differences,** | **9 and 10** |
| Conclusion/Recommendation | **The conclusion suits the study findings, but stick to the findings only** | **11** |
| References | **Update all references older than 10 years to a more recent study. Some of your references are very old, number 1, 4, 10 and 16.** | **12, 13, 14** |

**Rating of the manuscript:** Use (1 = Excellent) (2 = Very Good) (3 = Average) (4 = Fair) (5 = poor)

| Originality | **3** |
| --- | --- |
| Contribution To The Field | **3** |
| Technical Quality | **3** |
| Clarity Of Presentation | **2** |
| Depth Of Research | **3** |

**Recommendation:** Please, mark with an X

| Accept As It Is |  |
| --- | --- |
| Minor Corrections |  |
| Moderate Revision | **x** |
| Major Revision |  |
| Reject (Give Reasons) |  |

**Additional Comment(s):** Add any addition comment here

**The major work will be on restructuring the abstract and background statement,**

**indicate how the sample size was arrived at.**

**Restructure a little the discussion to include your view of other findings**

**Update the references to include on studies younger than 10 years.**
